# Fertility preservation in transgender and non-binary adolescents and young adults

**DOI:** 10.1371/journal.pone.0265043

**Published:** 2022-03-11

**Authors:** Holly C. Cooper, Jin Long, Tandy Aye

**Affiliations:** 1 Department of Pediatric Endocrinology, Mary Bridge Children’s Hospital, Tacoma, Washington, United States of America; 2 Department of Pediatrics, Stanford University School of Medicine, Stanford, California, United States of America; University of Connecticut, UNITED STATES

## Abstract

Although 37.5–51% of transgender adults state they would’ve considered freezing gametes before gender-affirming therapy if offered and 24–25.8% of transgender adolescents express interest in having biological children, less than 5% of transgender adolescents have opted for fertility preservation. We sought to assess fertility preservation utilization in our multidisciplinary adolescent gender clinic. We also aimed to identify fertility preservation utilization and interest among non-binary adolescents and young adults. A retrospective review was conducted of patients seen in the Stanford Pediatric & Adolescent Gender Clinic from October 2015 through March 2019 who were >10 years of age at initial visit. All individuals with documented discussion of fertility preservation were offered referral for formal fertility preservation consultation but only 24% of patients accepted. Only 6.8% of individuals subsequently underwent fertility preservation (n = 9). Transfeminine adolescents are more likely to pursue fertility preservation than transmasculine adolescents (p = 0.01). The rate of fertility preservation in non-binary adolescents did not significantly differ from those in transfeminine adolescents (p = 1.00) or transmasculine adolescents (p = 0.31). Although only one non-binary individual underwent fertility preservation, several more expressed interest with 36% accepting referral (n = 4) and 27% being seen in consultation (n = 3). Despite offering fertility preservation with designated members of a gender clinic team, utilization remains low in transgender adolescents. Additionally, non-binary adolescents and their families are interested in fertility preservation and referrals should be offered to these individuals. Further studies and advocacy are required to continue to address fertility needs of transgender adolescents.

## Introduction

Gender variance, also referred to as gender expansiveness, is a situation in which a person’s sex assigned at birth is incongruous with the gender with which they identify [1, 2]. Gender dysphoria is the condition of psychological distress associated with gender variance [1, 2]. In addition to transfeminine individuals who were assigned male at birth but identify as female and transmasculine individuals who were assigned female at birth but identify as male, there is increasing recognition of a set of individuals who identify outside of this binary system [1, 3]. Although the specifics of the gender identities vary considerably within this group, they are often categorized together as non-binary as they all identify outside of the traditional dichotomous system of male versus female. In the United States, 0.7% of patients age 13–17 years, or an estimated 150,000 adolescents, identify as transgender based on a published report from 2017 [4, 5]. With increased awareness of gender variance and transgender and non-binary people, these individuals are receiving easier access to care and earlier hormonal interventions. These gender-affirming therapies include puberty-suppressing gonadotropin-releasing hormone agonists (GnRHa) and sex hormone therapy (estrogen for those desiring feminine secondary sex characteristics and testosterone for those desiring masculine secondary sex characteristics). Gender-affirming therapies may also include surgical interventions such as top surgery (breast augmentation or chest reduction), and gonadectomy. Although these therapies are potentially available to all non-binary and transgender individuals, an individual may decide to pursue some or all of these treatment modalities depending on their specific goals [1, 6].

As gender-affirming hormone therapy in adolescents increases, fertility preservation has become an area of increasing concern for parents, adolescents, and their gender healthcare providers due to the potential for irreversible changes in fertility. Long-term use of these gender-affirming sex hormones may result in infertility though it remains somewhat uncertain what duration of hormonal treatment produces irreversible effects [7–11]. Research in the transgender adult population demonstrates that 51% of transfeminine adults and 37.5% of transmasculine adults would have considered freezing gametes (sperm or ova) before gender-affirming medical therapy had it been offered [12, 13]. New guidelines from the World Professional Association of Transgender Health (WPATH), American Society for Reproductive Medicine, and Endocrine Society include counselling and discussion of options for gamete preservation prior to initiation of hormonal treatments [1, 3, 14]. More recent studies of attitudes toward fertility in transgender adolescents demonstrate lower rates of interest than those of adults with only about one quarter expressing a desire for biological children [15, 16]. Despite increasing counselling on fertility preservation, two recent studies in the United States (US) report gamete preservation in <5% of their pediatric and adolescent transgender patients [17, 18]. In a 2019 Canadian study, similarly low uptake of fertility preservation was reported with zero out of 79 transgender youths undergoing fertility preservation [19]. However, higher rates of fertility preservation were seen in transfeminine adolescents in the Netherlands where ~34.6% attempted fertility preservation [20].

Although prior studies assessing fertility preservation utilization in adolescents and young adults have primarily focused on transfeminine and/or transmasculine individuals, there are some data in non-binary populations which demonstrate a strong interest in fertility discussions. In a survey conducted of Australian non-binary and transgender adults, 95% felt that fertility preservation should be offered to all transgender and non-binary people and 7% reported having undergone a fertility preservation procedure [21]. Belgian non-binary adults have similarly expressed interest, with 9.1%-37.5% of individuals surveyed reporting a current or future desire for biological children [22, 23]. Other studies in gender expansive adolescents and adults have included non-binary individuals as part of their study population but discussions have not focused on this population in particular, instead either grouping them with their sex assigned at birth or with the study population as a whole [19, 24–26].

This study sought to assess the fertility preservation utilization rate when services were offered as part of a multidisciplinary pediatric gender clinic team, compared to previous studies when external referrals were provided. In addition, we aimed to investigate how non-binary individuals utilize fertility preservation as no study to date has assessed fertility preservation practices in non-binary adolescents and young adults.

## Methods

This review of electronic medical records was conducted of all patients seen in the Stanford Pediatric & Adolescent Gender Clinic from October 2015 through March 2019 who were >10 years of age at time of initial visit. Prior to initiation of data collection, the protocol was submitted to the Institutional Review Board and granted exemption for formal protocol review and approval. Individuals were excluded from the review if they were pre-pubertal (sexual-maturity rating or Tanner stage 1), not interested in beginning gender-affirming therapy, or had previously initiated gender-affirming therapy with another gender healthcare provider. Gender-affirming therapy was defined as the use of GnRHa and/or gender-affirming sex hormones. Information obtained from chart review included patient age at clinic visit, sex assigned at birth, affirmed gender, outcome of fertility preservation counselling, and reasons for declining fertility preservation if documented.

### Clinic structure

The Stanford Pediatric & Adolescent Gender Clinic is a single, multidisciplinary clinic team which consists of on-site members specializing in pediatric endocrinology, adolescent medicine, gynecology, urology, psychology, and social work. In addition to these on-site members, there are also designated team members, including a Urologist specializing in male infertility and a Reproductive Endocrinology and Infertility (REI) specialist, located one floor down from the Gender clinic. If patients express interest in fertility preservation, a referral is placed to this specific adolescent fertility and REI specialist or our designated infertility urologist whose clinics are located in the same building.

### Statistical analysis

Descriptive statistics were used to summarize the included patients and the subgroups (transfeminine, transmasculine and non-binary), with frequencies for categorical variables and mean plus range for continuous variables. The comparisons among subgroups were performed using Fisher’s exact tests with a significant level as p < 0.05. All statistical analyses were conducted using SAS 9.4 (SAS Institute, Cary, NC).

## Results

### Overall fertility preservation

**Fig 1:**
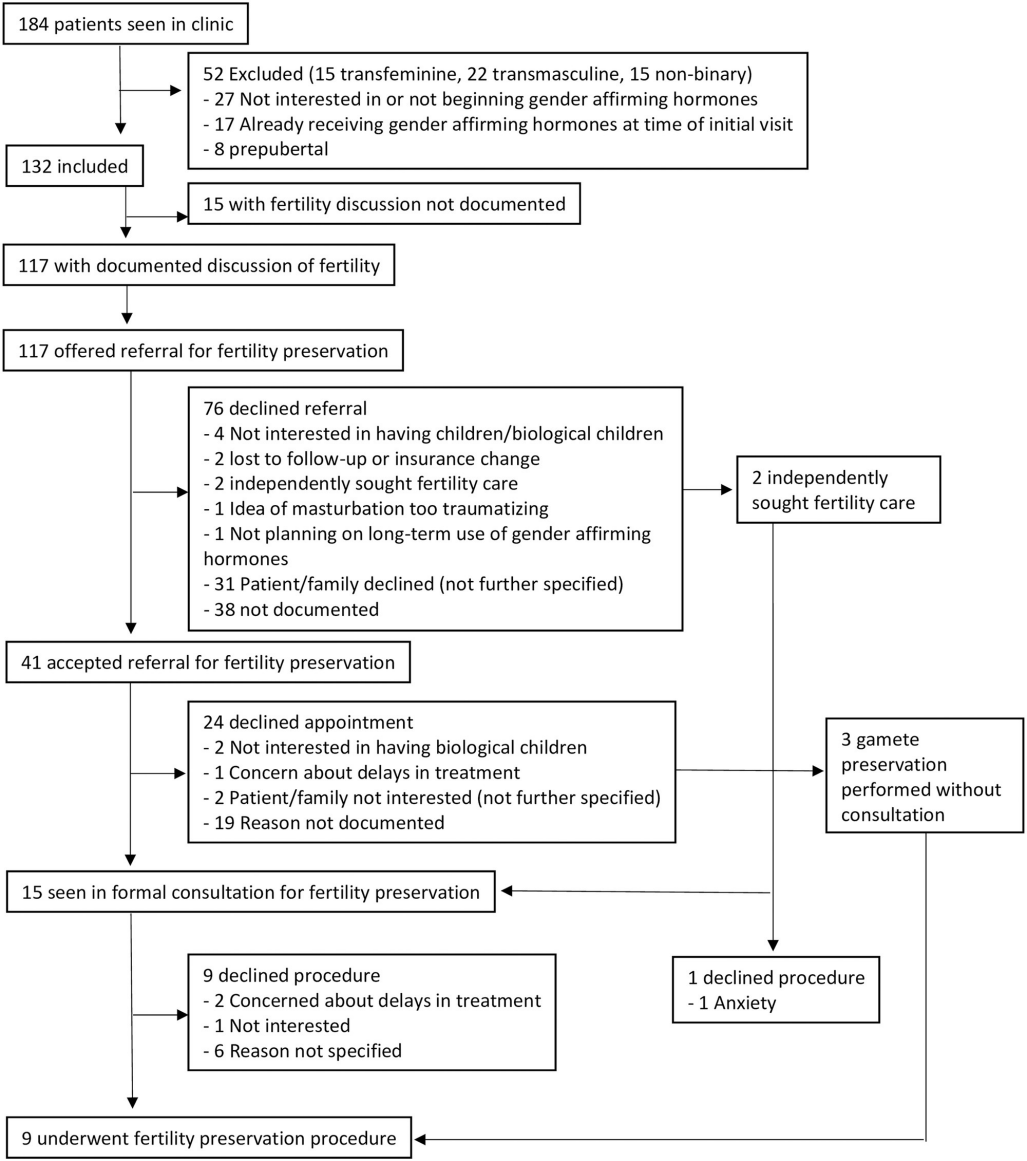
Study flow diagram. Outlines total individuals in study and participation in each step of fertility preservation pathway.

**Fig 2:**
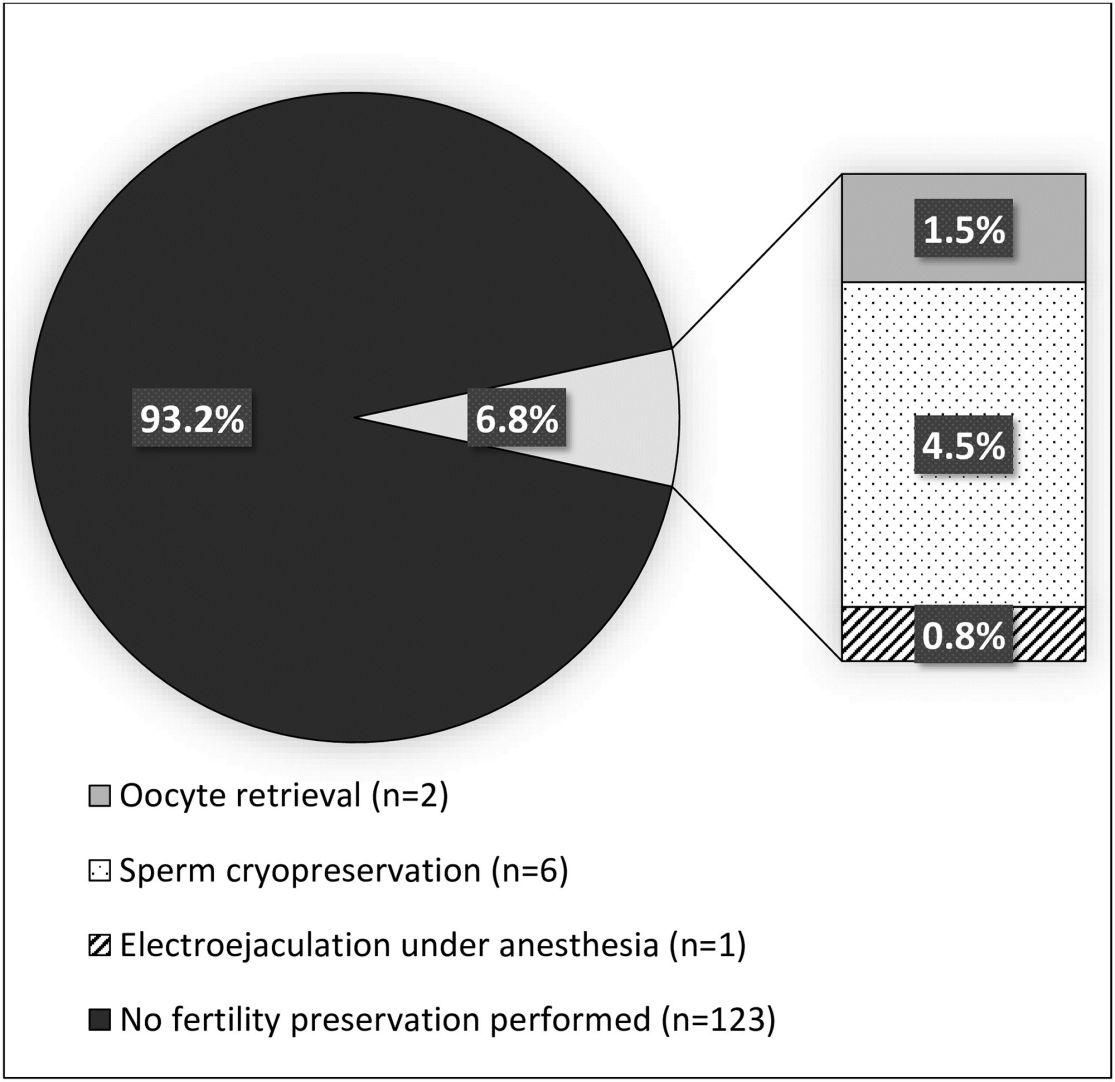
Overall fertility preservation rates. Fertility preservation rates in the Stanford Pediatric & Adolescent Gender Clinic from October 2015 through March 2019 (total n = 132).

**Fig 3:**
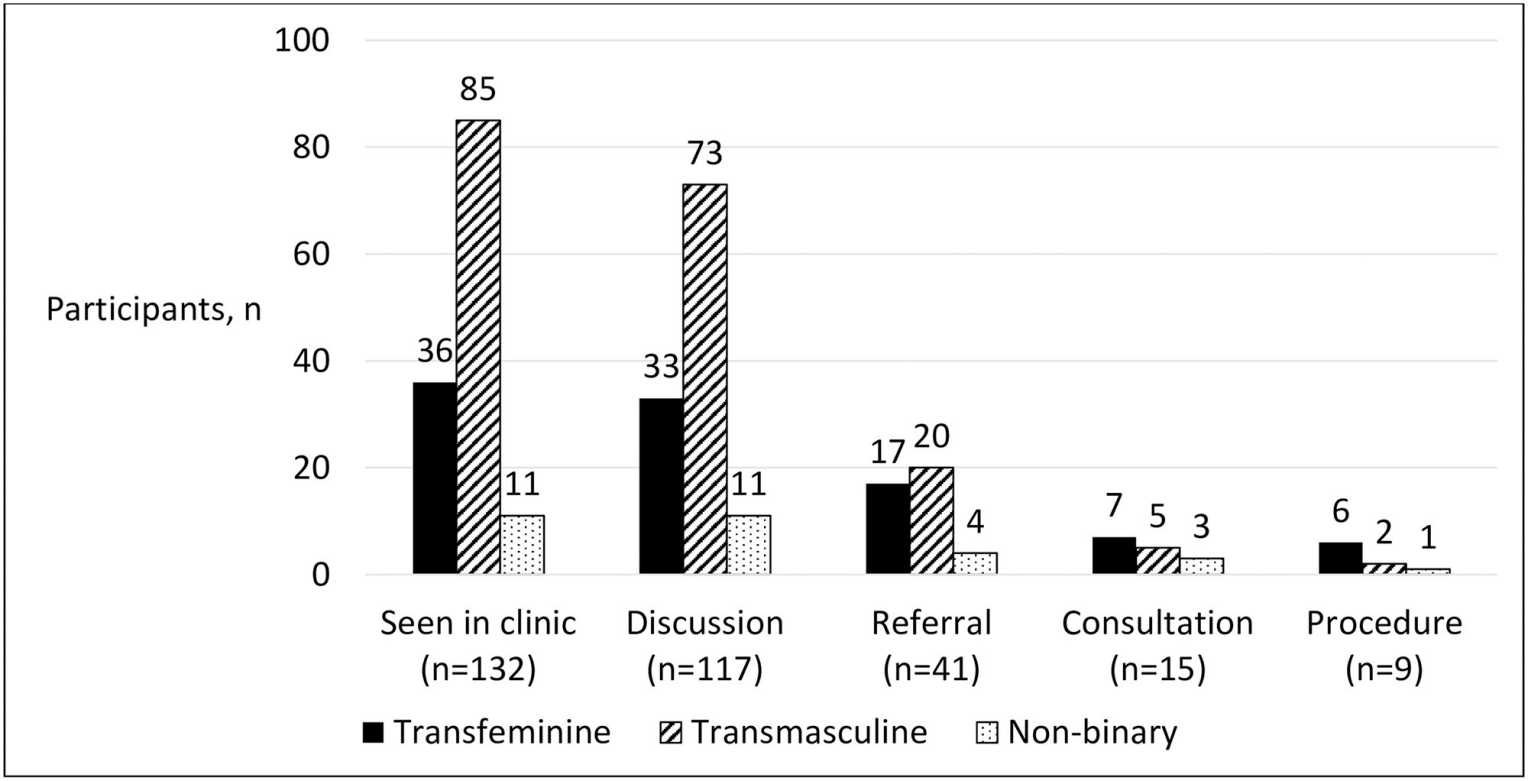
Fertility preservation consultation and utilization by gender identity. Patients are classified as “Seen in clinic” if they met inclusion criteria. Those that have documented discussion of fertility preservation are included in “Discussion” and those who accepted referral in “Referral.” If patients were seen in formal consultation for fertility preservation or underwent a fertility preservation procedure, they are included in “Consultation” and “Procedure,” respectively. Transfeminine patients are assigned male at birth but identify as female while transmasculine patients are assigned female at birth but identify as male. Non-binary patients do not identify as exclusively male or female and may have been assigned either male or female at birth. Numbers above bars indicate counts (n) within each category with a total of 132 patients.

Of the 184 eligible patients seen during the study period, 132 met inclusion criteria (Fig 1). Of the 52 patients who were excluded from the study, 8 were prepubertal, 27 were not interested in or not beginning gender-affirming therapy, and 17 had already been prescribed gender-affirming hormone therapy by another gender healthcare provider prior to their initial visit. Demographics of study participants are summarized in Table 1 and fertility preservation utilization is summarized in Figs 2 and 3.

**Table 1:** Baseline characteristics and demographics.

		Transfeminine	Transmasculine	Non-binary	Total, n
Gender identity, n		36	85	11	132
Sex assigned at birth, n	Male	36	-	4	40
	Female	-	85	7	92
Age at initial visit	10–13.99 years, n	8	20	0	28
	14–18 years, n	22	57	8	87
	> or = 18 years, n	6	8	3	17
	Mean age in years	15.7	15.5	17.2	-
	Age range in years	11.3–21.3	10.6–20.6	15.3–21.4	-
Insurance, n	Public Insurance	9	25	1	35
	Private Insurance	27	60	10	97

This table includes baseline characteristics of the 132 patients included in this study including sex assigned at birth, gender identity, mean age (and age range) at initial gender clinic visit, and insurance type.

All patients received fertility counseling as part of the written and verbal consent for gender-affirming hormone therapy though only 89% of the study patients (n = 117) had documentation of this discussion in their medical records, including documented offer of referral to fertility specialist. Only 31% of patients (n = 41) accepted the referral for formal consultation and of these individuals, only 36.6% of them (n = 15) were seen for an appointment. A total of 9 patients ultimately underwent fertility preservation (2 oocyte and 7 sperm), making the overall rate 6.8%. Only 12 individuals had documentation of reasons for declining fertility preservation which included lack of interest in having children and/or biological children (n = 6, 50%), concern about delay in treatment or need for further pubertal progression prior to fertility preservation (n = 3, 25%), possible plans for future discontinuation of gender-affirming hormones (n = 2, 16.7%), and discomfort with the idea of masturbation for semen sample collection (n = 1, 8.3%). Two individuals also expressed a potential desire to stop sex hormone therapy in the future for fertility preservation or to have children later in life.

### Fertility preservation by gender identity

Fertility preservation by gender identity is depicted in Fig 3. Out of 36 transfeminine adolescents and young adults, 17 patients accepted referral for fertility preservation and 6 patients were seen for formal consultation in urology clinic. A total of 6 transfeminine patients (~16.7% of all transfeminine patients) underwent sperm cryopreservation, all via standard semen sample collection through masturbation. Of the 85 transmasculine adolescents and young adults who were seen in clinic, 20 accepted the referral for formal consultation and 5 were eventually seen for formal consultation with REI. Ultimately only 2 of these patients (~2.3%) underwent oocyte retrieval and cryopreservation.

A total of 11 non-binary patients met inclusion criteria for this study. All of these individuals had documented discussion of fertility preservation but only 4 accepted referral for fertility preservation. Three non-binary patients were seen in formal consultation for fertility preservation and only 1 patient (~9.1%) ultimately underwent a fertility preservation procedure. Due to difficulty producing a sample through masturbation, this patient underwent electroejaculation under anesthesia to obtain a sample for sperm cryopreservation.

In this study, transfeminine adolescents and young adults were significantly more likely to pursue fertility preservation than transmasculine adolescents (p = 0.01). The rate of fertility preservation in non-binary individuals did not significantly differ from those in transfeminine individuals or transmasculine individuals (p = 1.00 and p = 0.31, respectively).

## Discussion

Despite the inclusion of designated gender care team members who specialize in fertility preservation, utilization among transgender adolescents in the US remains low at 6.8% in this study population (Fig 2). Only 24% of patients with documented discussion of fertility accepted a referral for fertility preservation and 6.8% underwent fertility preservation (2 oocyte and 7 sperm). Only one non-binary individual underwent fertility preservation but several more expressed interest in fertility preservation by accepting a referral or being seen in consultation. However, the overall number of non-binary individuals in this study was low (n = 11) as more than half of the individuals seen in clinic were excluded because they were not starting gender-affirming hormonal treatments (15 excluded from initial group of 26).

The relatively low overall rate of fertility preservation in our study, 6.8%, is consistent with prior studies in adolescents from US and Canada-based studies [17–19], despite the increased convenience of providing fertility preservation services by designated members of the Gender Clinic team. Interestingly, in a recent report by Brik, et al. from a Netherlands clinic, the rate of fertility preservation was considerably higher at 34% [20]. This Dutch study only assessed fertility preservation utilization in transfeminine adolescents (n = 35) which can account for some of the increase though rates from studies for transfeminine adolescents remain considerably lower at 9–14% [17, 18]. It was postulated in the Dutch study that the increased insurance coverage of fertility preservation for transgender individuals in the Netherlands explains the difference in fertility preservation rates since cost has been consistently reported as a barrier in US studies [17, 18, 24, 27]. Even with insurance, fertility preservation for transgender adolescents and young adults in the US typically requires large out-of-pocket expenses including not only procedures but also years of cryopreservation and for transmasculine individuals can cost tens of thousands of US dollars depending on the method of oocyte retrieval [27, 28]. It was also suggested that differing perceptions on adoption in the two countries could have led more Dutch transfeminine adolescents to undergo fertility preservation as only 13% of patients reported interest in adopting children in the future compared to US and Canadian studies where 52–80% reported interest in adoption [16, 19, 20, 29].

As in prior studies, the rates of fertility preservation were higher in transfeminine adolescents compared to transmasculine adolescents (16.7% and 2.3% in this study, respectively vs. 9–14% and 0–1.2% in prior studies) [17, 18]. This study is the first to report on fertility preservation utilization in the non-binary adolescent and young adult population and to compare it to transfeminine and transmasculine adolescents and young adults. Only 1 non-binary patient underwent fertility preservation but due to the relatively lower proportion of these patients in our clinic who met inclusion criteria for this study, this represents a rate of ~9%. Depending on the goals of non-binary individuals, they may or may not pursue full gender affirmation therapy which can include hormone therapy, top surgery (breast augmentation or chest reduction), and gonadectomy and thus may not have the same risk of infertility as transmasculine and transfeminine patients. Regardless, it is clear from the rates of referral and consultation (36% and 27%, respectively) that non-binary individuals who were interested in gender-affirming hormone therapy also have an interest in fertility preservation and deserve the same counselling and options as their binary counterparts.

Despite the creation of a multidisciplinary gender clinic with designated fertility preservation providers, challenges navigating the system remain a barrier to fertility preservation in our transgender population. One patient had their referral to fertility preservation denied as they had already updated their name and gender marker (male vs. female vs. non-binary) and the referral was inappropriately flagged as in error. Although this error was remedied with resubmission of the referral, it created a delay in approval. Continued education in workflow and streamlining of the referral process is required to ensure effective and complete healthcare is provided to transgender and gender non-conforming individuals.

Limitations of this study include its retrospective design and relatively small and unevenly distributed study population. Additionally, if patients independently sought fertility preservation and did not inform their gender healthcare provider, they may not have been included in total numbers. These reported data also do not include 1 patient who had already undergone fertility preservation prior to initial clinic visit. Additionally, the reasons for declining fertility preservation were obtained through chart review, rather than directed interview, and thus likely represent incomplete data.

The majority of currently available fertility preservation techniques require the child undergo a substantial amount of pubertal progression of the “noncongruent” gender, which includes some irreversible changes, before gamete preservation is possible. Whole gonad cryopreservation with in-vitro activation is an area of ongoing research which may potentially allow fertility preservation without requiring pubertal progression though this is not currently a clinically viable option [30–32]. Furthermore, the process of fertility preservation can result in increased gender dysphoria in individuals assigned female at birth due to the nature of the process which requires transvaginal ultrasounds and genital exams [33] and in individuals assigned male at birth who are uncomfortable with masturbation for semen sample collection [20]. Although progress has already been made to unite the fields of pediatric onco-fertility and pediatric transgender fertility, thereby increasing options and knowledge of pediatric and adolescent fertility [34], additional research into alternative fertility preservation techniques in this field is required to minimize dysphoria while promoting future fecundity.

## Conclusion

Despite increased attention in recent years, the field of pediatric and adolescent transgender fertility remains an understudied and under-discussed area of medicine. Fertility preservation utilization in adolescents seeking gender-affirming hormone therapy is low across all gender identities despite prior reports of high levels of interest in adult transgender individuals. Non-binary adolescents, like their transfeminine and transmasculine counterparts, have an interest in fertility preservation and should be offered the same counseling and opportunities. Continued work in the field is required to increase fertility preservation options in this population. Ongoing nationwide advocacy is also required to promote insurance coverage of fertility preservation to reduce the impact of cost on decisions about fertility.

## Supporting information

S1 Data (XLSX)
